# The Medical Expert System in Germany

**Published:** 1879-08

**Authors:** 


### THE MEDICAL EXPERT SYSTEM IN GERMANY.

In Germany alone has medical jurisprudence received a proper estimate of its worth. It was introduced into the German Universities at their foundation, and made a part of medical education. The expert is made a government officer, holding his position because of his peculiar skill and fitness. The present organization is the result of continuous legislation upon the subject since the early part of the sixteenth century. It consists entirely of medico-legal officials, and is divided into four divisions. The county physician and county surgeon constitute the first, the county tribunal.

They are required by law to be thoroughly educated in medicine, surgery and obstetrics, and to have special training in medical jurisprudence. This training is certified to by examination before a supreme medical commission. The duties of the county tribunal are twofold. It is incumbent on them to investigate all medico-legal questions referred to them by the courts. They also perform the duties which in this country are performed by the coroner. Their appointment is continuous and they owe it to neither party, but to the State. They make personal investigation of the facts in every case. Power is given them to take testimony.. They may call experts as counsel if they wish, but these experts must have certificates, received from State examinations, of their skill in the specialty whose principles it is desired to apply. The facts and proofs and the conclusions arrived at by the expert tribunal, are then shaped into a written report, which is carefully preserved, along with the report of the subsequent proceedings, and so far as the court making the reference is concerned, this report is conclusive, but the physician and surgeon may disagree, or the parties interested may be dissatisfied. To meet these cases, an appeal is made to the second division of this organization. This division consists of a medical commission of from four to six members, and is somewhat analagous to our district courts. One such commission exists in every province. If dissatisfaction exists with the decision of this tribunal, an appeal lies to the third division. This is the supreme medical commission, comprising men of national reputation. In the fourth division we find the Minister of medical and sanitary affairs, who presides over the entire department of State medicine.—*Pacific Medical and Surgical Journal.*

